# Retraction: Effectiveness of medical ozone injections into the intervertebral disc on relieving lumbosacral pain–a systematic review and meta-analysis

**DOI:** 10.3389/fpain.2026.1972267

**Published:** 2026-08-27

**Authors:** Frontiers Editorial Office

The journal retracts the 27 October 2025 article cited above.

Following publication, concerns were raised regarding the scientific validity of the article. An investigation was conducted in accordance with Frontiers' policies.

It was found that the complaints were valid and that the article does not meet the standards of editorial and scientific soundness for Frontiers in Pain Research; therefore, the article has been retracted.

This retraction was approved by the Chief Editors of Frontiers in Pain Research and the Chief Executive Editor of Frontiers. The authors agree to this retraction.

Frontiers would like to thank the concerned reader who contacted us regarding the published article.

